# Fine Particulate Matter Exposure Alters Pulmonary Microbiota Composition and Aggravates Pneumococcus-Induced Lung Pathogenesis

**DOI:** 10.3389/fcell.2020.570484

**Published:** 2020-10-26

**Authors:** Yu-Wen Chen, Shiao-Wen Li, Chia-Der Lin, Mei-Zi Huang, Hwai-Jeng Lin, Chia-Yin Chin, Yi-Ru Lai, Cheng-Hsun Chiu, Chia-Yu Yang, Chih-Ho Lai

**Affiliations:** ^1^Department of Microbiology and Immunology, Graduate Institute of Biomedical Sciences, College of Medicine, Chang Gung University, Taoyuan, Taiwan; ^2^Molecular Medicine Research Center, Chang Gung University, Taoyuan, Taiwan; ^3^Department of Otolaryngology-Head and Neck Surgery, School of Medicine, China Medical University and Hospital, Taichung, Taiwan; ^4^Division of Gastroenterology and Hepatology, Department of Internal Medicine, Shuang-Ho Hospital, New Taipei, Taiwan; ^5^Division of Gastroenterology and Hepatology, Department of Internal Medicine, School of Medicine, College of Medicine, Taipei Medical University, Taipei, Taiwan; ^6^Molecular Infectious Disease Research Center, Department of Pediatrics, Chang Gung Memorial Hospital, Linkou, Taiwan; ^7^Department of Otolaryngology-Head and Neck Surgery, Chang Gung Memorial Hospital, Taoyuan, Taiwan; ^8^Department of Nursing, Asia University, Taichung, Taiwan

**Keywords:** PM_2.__5_, pulmonary inflammation, microbiota, pneumococcus, pathogenesis

## Abstract

Exposure to fine particulate matter (PM) with aerodynamic diameter ≤2.5 μm (PM_2.__5_) is closely correlated with respiratory diseases. Microbiota plays a key role in maintaining body homeostasis including regulation of host immune status and metabolism. As reported recently, PM_2.__5_ exposure causes microbiota dysbiosis and thus promotes disease progression. However, whether PM_2.__5_ alters pulmonary microbiota distribution and aggravates bacteria-induced pathogenesis remains unknown. In this study, we used mouse experimental models of PM_2.__5_ exposure combined with *Streptococcus pneumonia* infection. We characterized the airway microbiota of bronchoalveolar lavage fluid (BALF) by sequencing the 16S rRNA V3–V4 amplicon on the Illumina MiSeq platform, followed by a combination of bioinformatics and statistical analyses. Shannon-diversity index, observed ASVs, and Fisher’s diversity index indicated that microbiota richness was significantly decreased in the mice treated with either PM_2.__5_ or pneumococcus when compared with the control group. The genera *Streptococcus*, *Prevotella*, *Leptotrichia*, and *Granulicatella* were remarkably increased in mice exposed to PM_2.__5_ combined with pneumococcal infection as compared to mice with pneumococcal infection alone. Histopathological examination exhibited that a more pronounced inflammation was present in lungs of mice treated with PM_2.__5_ and pneumococcus than that in mouse groups exposed to either PM_2.__5_ or pneumococcal infection alone. Our results demonstrate that PM_2.__5_ alters the microbiota composition, thereby enhancing susceptibility to pneumococcal infection and exacerbating lung pathogenesis.

## Introduction

Air pollution is the cause and aggravating factor of many respiratory diseases, including respiratory infections, allergies, asthma, and chronic obstructive pulmonary disease (COPD) (Feng et al., 2016). Particles and gases are the major air pollution components that invade into the human lungs during respiration (Falcon-Rodriguez et al., 2016). These particles with an aerodynamic diameter ≤2.5 μm (PM_2.__5_) can penetrate and deposit in the bronchi and can affect human health, leading to severe consequences such as respiratory and cardiovascular diseases (Madrigano et al., 2013; Habre et al., 2014; Guo et al., 2018; Zhao et al., 2020). More importantly, PM_2.__5_ exposure has been linked to increased cardiopulmonary diseases and other related mortalities (Gehring et al., 2015; Pope et al., 2019), indicating that they pose a potent public health risk.

Pneumococcal pneumonia, caused by *Streptococcus pneumoniae*, which is a Gram-positive bacterium with the shape of diplococci. Pneumococcal infection often occurred in children with high morbidity and mortality, and the incidence of community-acquired pneumonia increases with age (Neupane et al., 2010). Notably, air pollution is closely related to the occurrence of community-acquired pneumonia (Shears et al., 2020). However, the mechanism how particulate matter influences the host defenses against pneumococcal infection remains to be elucidated.

The bacterial community plays an important role in host physiology, such as defenses against pathogens, regulation of immune response, and modulation of metabolism (Shapiro et al., 2014; Liu et al., 2015). Alterations in the bacterial dynamic ecosystem can directly influence immune homeostasis and aggravate inflammatory diseases (Rakoff-Nahoum et al., 2004; Pascal et al., 2018). Recent studies have shown that PM_2.__5_ exposure causes bacterial community dysbiosis and exacerbate disease development (Mariani et al., 2018; Qin et al., 2019; Wang et al., 2019). However, whether PM_2.__5_ influences respiratory microbiota distribution and facilitates bacterial infection remains unknown.

High-throughput 16S rRNA sequencing has been widely utilized to analyze the microbiota community in recent years. PM_2.__5_ exposure is reported to alter bacterial composition in the nasal pathway (Mariani et al., 2018), airway (Qin et al., 2019; Wang et al., 2019), and gut (Mutlu et al., 2018; Wang et al., 2018). However, the mechanisms by which PM_2.__5_ influences the microbiota community in lungs and enhances pathogenic bacterial infection in respiratory tract are unclear. For this purpose, a murine experimental model was established by exposing the mice to PM_2.__5_ for 3 weeks followed by pneumococcal infection. The BALF microbiota composition and altered metabolism in response to PM_2.__5_ exposure were analyzed. Our results indicated that PM_2.__5_ influences the microbiota composition that associated with pneumococcus-induced pulmonary pathogenesis.

## Materials and Methods

### Cell and Bacterial Culture

Macrophage cell line RAW264.7 (ATCC TIB-71) cells were cultured in Dulbecco’s Modified Eagle Medium (DMEM) supplemented with 10% complement-inactivated fetal bovine serum (FBS). Pneumococcus (*Streptococcus pneumoniae* strain TIGR4, ATCC BAA-334) was cultured on blood agar plates (Becton Dickinson, Sparks, MD, United States) as described previously (Lee et al., 2018). The bacteria were refreshed in Todd Hewitt Broth (Becton Dickinson) for 3 h and prepared for conducting the experiments in murine.

### Macrophage Killing Assay

RAW264.7 (5 × 10^5^) cells were suspended with PM_2.__5_ (5 or 20 μg/ml) and incubated at 37°C for 2 h. The cells were infected with pneumococcus at multiplicity of infection (MOI) of 100 for 30 min as described previously (Thevaranjan et al., 2018). The uninfected bacteria were removed, and the supernatant was collected from the following each 15 min and cultured on blood agar plates. Visible colony-forming units (CFU) were calculated, and macrophage killing activity was determined.

### Animal Study

**FIGURE 1:**
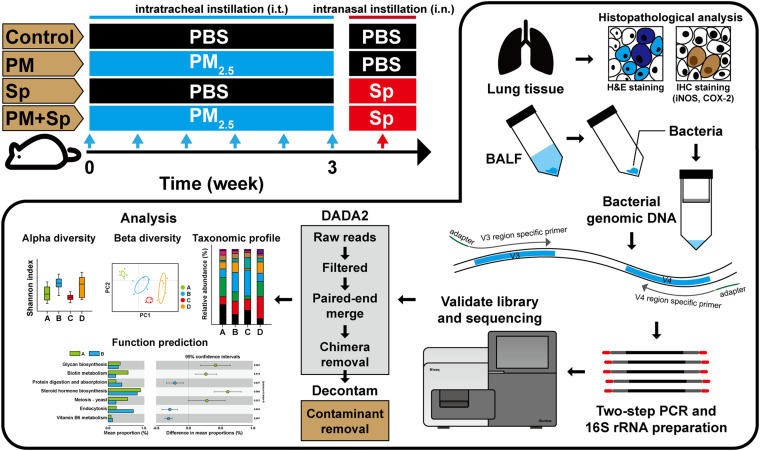
Experimental design of the animal study. Mice were intratracheally administered PM_2.__5_ (for a total of 200 μg) and then pneumococcus-infected (1 × 10^8^) via intranasal instillation. After pneumococcal infection for 48 h, the mice were euthanized. Their BALF were then prepared for microbiota analysis, and lung tissues were subjected to histopathological examination.

Male BALB/c mice (aged 6 weeks) were purchased from the National Laboratory Animal Center (Taipei, Taiwan). The animal studies were performed in accordance with the Animal Care and Use Guidelines for Chang Gung University under a protocol approved by the Institutional Animal Care Use Committee (IACUC Approval No.: CGU16-019). Three mice are housed in each cage and kept under normal conditions (21 ± 2°C and in a 12/12-h dark-light cycle) with sterile drinking water, feed, litter, and cages. Particulate matter with diameter smaller than 2.5 μm (PM_2.__5_) (RM8785) was purchased from the National Institute of Standards and Technology (Gaithersburg, MD, United States), as described previously (Klouda et al., 2005). Mice were divided into four groups for the treatments with PBS (control), PM_2.__5_, pneumococcus, and PM_2.__5_ + pneumococcus (six mice each group), respectively. PM_2.__5_ was administered by intratracheal (i.t.) instillation twice per week (six times for 3 weeks and for a total of 200 μg) (Figure 1). Mice were placed in the chambers for 4 days resting and then infected with pneumococcus by intranasal (i.n.) injection (1 × 10^8^ CFU/10 μl). After infection for 48 h, the mice were euthanized and the BALF (*n* = 4) and lungs (*n* = 2) were prepared as described previously (Lin et al., 2014). During the experimental procedure, one mouse in the control treatment and one mouse in the pneumococcal infection group died and were excluded in the following studies. A total of 14 BALF samples were used for sequencing, including the groups control (*n* = 3), PM_2.__5_ (*n* = 4), pneumococcus (*n* = 3), and PM_2.__5_ + pneumococcus (*n* = 4).

### Histopathological Analysis

Lung tissues isolated from mice were washed with PBS and prepared for hematoxylin–eosin (H&E) and immunohistochemistry (IHC) staining as described previously (Chen et al., 2018). The lung sections were stained with the COX-2 and iNOS, respectively. The stained tissues were observed and examined using a microscope (AXIO IMAGER M2, Carl Zeiss, Germany) by a histopathologist with the scoring evaluation (Lin et al., 2014): 0, normal lung tissue; 1, slight erythrocyte infiltration (interstitium/parenchyma) and slight cell infiltration around the bronchi; 2, obvious erythrocyte infiltration, injured interstitial lung, and slight cell infiltration around the bronchi; 3, substantial erythrocyte infiltration which diffused the interstitium/parenchyma and caused the lung inflammation; and 4, severe inflammation with substantial erythrocyte/immune cell infiltration and obvious thickening bronchial wall.

### Genomic DNA Extraction

The total bacterial genomic DNAs of BALF were extracted using the QIAamp DNA Microbiome Kit (Qiagen, Germantown, MD, United States) as per manufacturer’s instructions as described previously (Yang et al., 2018). Briefly, the AHL buffer was used to lyse host cells and the nucleic acids were digested, followed by removing the host DNA. ATL buffer was added to the bacterial cells in a pathogen lysis tube L and vortexed using a TissueLyser LT. The bacterial genomic DNA was then eluted using nuclease-free water and stored at −80°C until the preparation of sequencing libraries.

### 16S rRNA Sequencing

The bacterial 16S rRNA V3–V4 regions were amplified by PCR using primers as described previously (Yang et al., 2018). There was no product generated on reagent-only controls after the PCR amplification. Illumina adaptor overhang nucleotide sequences were added to these gene-specific sequences. The sequences of primers are forward: 5’-TCGTCGGCAGCGTCAGATGTGTATAAGAGACAGCCTACG GGNGGCWGCAG-3’; and reverse: 5’-GTCTCGTGGGCTCGG AGATGTGTATAAGAGACAGGACTACHVGGGTATCTAA TCC-3’ (Yang et al., 2018). Each PCR reaction mixture included bacterial genomic DNA (20 ng), aliquots of both forward and reverse primers (1 μM), and 1 × KAPA HiFi Hotstart Ready Mix. The first PCR program was performed with the following cycling: initial denaturation at 95°C for 3 min, followed by 30 cycles of denaturation at 95°C for 30 s, annealing at 55°C for 30 s, and extension at 72°C for 30 s; and a final 72°C extension for 10 min. The first PCR products of the 16S V3–V4 amplicons (∼550 bp) were purified using AMPure XP beads (Beckman Coulter, Indianapolis, IN, United States) and subjected to index PCR. Each index PCR reaction mixture included the purified first PCR products, Nextera XT index primer 1 and primer 2, and 1 × KAPA HiFi Hotstart Ready Mix. The index PCR program was performed with the following cycling: initial denaturation at 95°C for 3 min; 8 cycles of denaturation at 95°C for 30 s, annealing at 55°C for 30 s, extension at 72°C for 30 s, and a final extension at 72°C for 5 min. The index PCR products were purified by AMPure XP beads. The final amplicon libraries were validated using an HT DNA High Sensitivity LabChip kit (Caliper, PerkinElmer, MA, United States). The multiplexed amplified libraries were then sequenced using the Illumina MiSeq system (Illumina, San Diego, CA, United States). The raw sequence files supporting the findings of this article are available in the NCBI Sequence Read Archive under the BioProject ID PRJNA661979 (biosamples SAMN16072462 to SAMN16072475)^1^.

### Bioinformatic Analysis

The 16S rRNA V3–V4 sequencing reads were demultiplexed using MiSeq Reporter v2.6. The amplicon sequences were analyzed in accordance with MiSeq SOP (Kozich et al., 2013). 16S rRNA gene sequences were processed using the DADA2 pipeline to classify microbial constituents (Callahan et al., 2016). Following the DADA2 tutorial, paired-end sequences were separated through quality-filtering, dereplication, denoising, merging, and chimera removal. The quality-filtering step was performed with the filterAndTrim function in DADA2, and we used standard filtering parameters: maxN = 0, truncQ = 2, rm.phix = TRUE, and maxEE = 2. The truncate forward and reverse sequences were defined at positions 290 and 220, respectively, and the first 13 bases of each sequence were trimmed. A total of 1,521,645 sequences were used to construct amplicon sequence variants (ASVs), and ASVs comprising fewer than two reads were filtered from the dataset. The “Decontam” frequency method was used for contaminant removal by correlation with DNA concentration (Davis et al., 2018). As a result, 2,363 ASVs with quality-filtered sequences were obtained. A Naïve Bayes classifier was trained using the most recent available version of Silva (version 132) sequences for taxonomy assignment for each ASV through the assigned Taxonomy function (Wang et al., 2007). Comparison of species richness (observed ASVs, Shannon index, and Fisher’s index) between different groups was determined with phyloseq (McMurdie and Holmes, 2013). Beta diversity was performed using weighted Unifrac phylogenetic distance matrices (Lozupone and Knight, 2005). Two-dimensional PCoA plots to visualize bacterial community populations between two groups were generated with phyloseq. A PERMANOVA (α = 0.05) with 999 random permutations was performed to determine differences between groups using the function “Adonis” of the Vegan package. The microbial functionality profiles were predicted using PICRUSt2 (Douglas et al., 2020) to generate the Kyoto Encyclopedia of Genes and Genomes (KEGG) pathway based on 16S rRNA gene sequencing data. The predicted genes and their functions were aligned to the KEGG database, and the differences among groups were compared using STAMP (version 2.1.3). Two-side Welch’s *t*-test and Benjamini–Hochberg FDR correction were employed for comparisons of two groups.

### Statistical Analysis

Student’s *t*-test was employed to analyze the statistical significance of the experimental results between two groups. The statistical analysis was performed by using the SPSS program (version 18.0 for windows, SPSS Inc., Chicago, IL, United States). A *p-*value less than 0.05 was considered statistically significant. Differential abundance analysis was using the Kruskal–Wallis to detect main effect differences followed by the Wilcoxon rank-sum test for pairwise comparisons, with a *P* value cutoff of 0.05 and a linear discriminant analysis (LDA) score of 3.0 for identifying discriminative features by LEfSe with default parameters (Segata et al., 2011). We used the cladogram functionality of LEfSe to illustrate differences in BALF microbiota composition between with and without PM_2.__5_ treatments. In the cladogram, differences between groups are illustrated at phyla, class, order, family, and genus levels.

## Results

### PM_2.__5_ Exposure Influences Microbiota Profiles

**FIGURE 2:**
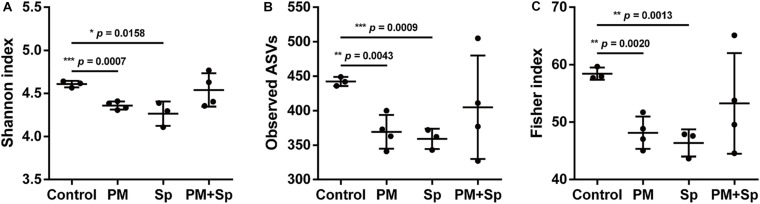
PM_2.__5_ alters BALF microbiota communities. **(A)** Shannon-diversity index, **(B)** observed ASVs, and **(C)** Fisher’s diversity index were conducted to assess microbiome diversity in BALF. Student’s *t*-test was used to analyze the statistical significance of the experimental results between two groups. *P < 0.05; **P < 0.01; ***P < 0.001. PM, PM_2.__5_ exposure; Sp, pneumococcal infection.

Firstly, we investigated whether PM_2.__5_ exposure affected the microbiota community in the respiratory tract. Mice were divided into four groups consisting of animals treated with either PBS (control), PM_2.__5_, pneumococcus (Sp), or PM_2.__5_ + pneumococcus (PM + Sp). After completing the treatment, mice were euthanized and BALF were collected for analyzing bacterial profiles. The bacterial genomic DNA were purified and analyzed using PCR amplification targeting 16S rRNA V3–V4 amplicons. The average number of raw reads per sample was 169,417 in 14 BALF samples (Supplementary Table 1). We obtained a total of 1,517,437 high-quality filtered reads, or 107,926.4 ± 21,089.7 reads per participant. The reads were constructed into 2,363 ASVs, and the sequence variants were used in the taxonomic analysis. The ASVs were classified into known taxa (22 phyla, 38 classes, 68 orders, 110 families, 285 genera, and 201 species) and unclassified groups. Taxonomic and phylogenetic information on the ASVs was shown in Supplementary Table 2. As an initial step, we compared the bacterial diversity and composition of BALF in mice subjected to different treatments. The BALF microbiota communities in PM_2.__5_-exposure mice showed a significantly lower value of the Shannon-diversity index than the control group (Figure 2A). In addition, the two other richness factors, observed ASVs and Fisher index, were also decreased in mice exposed to PM_2.__5_ when compared with the control group (Figures 2B,C). Similarly, the microbiota communities in the pneumococcus infection group were less diverse than those in the control group. Next, the beta-diversity was assessed using principal coordinate analysis (PCoA) to determine the microbiota diversity in different groups. According to the weighted Unifrac distance calculation performed on microbiota profiles followed by the Adonis test (*p* = 0.001 and *R*^2^ = 0.48), the PCoA plot showed that the BALF bacterial compositions with spatial separations were varied among different groups (Supplementary Figure 1). These results suggest that the BALF microbiota communities were divergent among the control, PM_2.__5_, and/or pneumococcal treatment groups.

### Alterations in Most Abundant Bacterial Taxa in PM_2.__5_-Treated Mice

**FIGURE 3:**
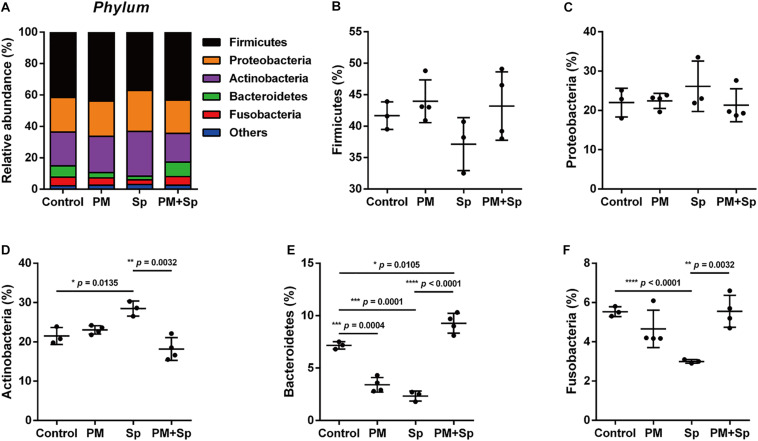
Relative abundance of bacterial phyla in mice exposed to PM_2.__5_ and pneumococcus. The bacteria taxonomic profiles at the phylum level in BALF microbiota from PM_2.__5_-treated (PM) and/or pneumococcus-infected (Sp) mice. **(A)** The top 5 most abundant phyla in the four groups were shown. Relative abundances of **(B)** *Firmicutes*, **(C)** *Proteobacteria*, **(D)** *Actinobacteria*, **(E)** *Bacteroidetes*, and **(F)** *Fusobacteria* in the BALF microbiota community were analyzed. Student’s *t*-test was used to analyze the statistical significance of the experimental results between two groups. *P < 0.05; **P < 0.01; ***P < 0.001.

The most abundant bacteria at the phylum and genus levels within groups were investigated. The dataset indicated a total of 6 phyla (frequency higher than 0.001 in four groups), and 5 of them accounted for 97% bacteria. In the control mice, the top five relatively abundant bacteria were *Firmicutes* (41.5 ± 2.6%), *Proteobacteria* (23.0 ± 1.9%), *Actinobacteria* (22.8 ± 3.7%), *Bacteroidetes* (5.5 ± 2.8%), and *Fusobacteria* (4.7 ± 1.0%) (Figure 3A). There was no significant difference in *Firmicutes* and *Proteobacteria* levels between the PM_2.__5_-exposed and pneumococcus-infected groups (Figures 3B,C). In pneumococcus-infected mice, *Actinobacteria* (28.5%, *p* = 0.0135) was increased but *Bacteroidetes* (2.3%, *p* = 0.0001) and *Fusobacteria* (3.0%, *p* < 0.0001) were decreased as compared with the control group (Figures 3D-F). In mice exposed to PM_2.__5_, *Bacteroidetes* (3.4%, *p* = 0.0004) and *Fusobacteria* (4.7%, *p* < 0.0001) were decreased. Noticeably, *Actinobacteria* (18.2%, *p* = 0.0032) was decreased, but both *Bacteroidetes* (9.3%, *p* < 0.0001) and *Fusobacteria* (5.5%, *p* = 0.0032) were significantly higher in mice exposed to PM_2.__5_ + pneumococcus than in the pneumococcus-infected group.

**FIGURE 4:**
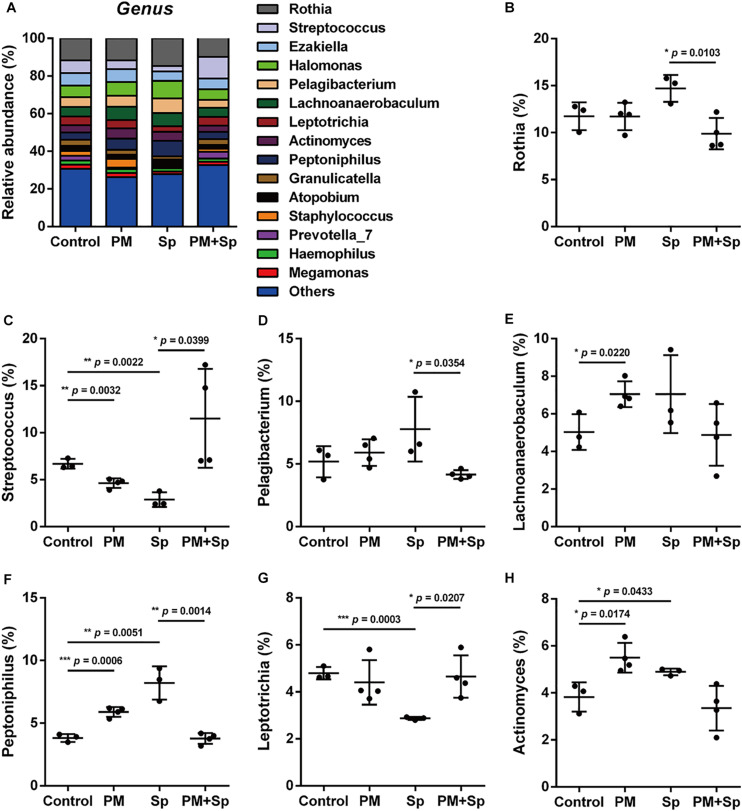
Relative abundance of bacterial genera in mice treated with PM_2.__5_ and pneumococcus. The bacteria taxonomic profiles at the genus level in BALF microbiome from PM_2.__5_-treated (PM) and/or pneumococcus-infected (Sp) mice. **(A)** The top 15 most abundant genera among four groups were shown. Relative abundances of **(B)** *Rothia*, **(C)** *Streptococcus*, **(D)** *Pelagibacterium*, **(E)** *Lachnoanaerobaculum*, **(F)** *Peptoniphilus*, **(G)** *Leptotrichia*, and **(H)** *Actinomyces* in BALF microbiota community were analyzed. Student’s *t*-test was used to analyze the statistical significance of the experimental results between two groups. *P < 0.05; **P < 0.01; ***P < 0.001.

The profiles of major microbiota at the genus level were further analyzed. Our data showed a total of 16 genera (all frequency higher than 0.01), of which the top 15 genera were identified (Figure 4A and Supplementary Figure 2). The 5 predominant genera were *Rothia* (12.0 ± 1.7%), *Halomonas* (7.1 ± 1.4%), *Streptococcus* (6.4 ± 3.2%), *Ezakiella* (6.0 ± 0.7%), and *Pelagibacterium* (5.8 ± 1.3%) in the control group. In the 15 genera of most relative abundance, 7 were significantly modulated among different treatment groups (Figures 4B–H and Supplementary Figure 2). In mice exposed to PM_2.__5_, 5 genera were altered, with an increase in 3 genera (*Lachnoanaerobaculum*, *Peptoniphilus*, and *Actinomyces*) (*p* < 0.05), and a decrease in 2 genera (*Streptococcus* and *Prevotella*) (*p* < 0.05). Compared to the pneumococcus-infected group, mice exposed to PM_2.__5_ + pneumococcus showed changes in 8 genera, with an increase in 4 genera (*Streptococcus*, *Leptotrichia*, *Granulicatella*, and *Prevotella*) (*p* < 0.05) and a decrease in the 4 genera (*Rothia*, *Pelagibacterium*, *Peptoniphilus*, and *Atopobium*) (*p* < 0.05). These results indicate that microbial abundance was altered in mice treated with either PM_2.__5_ and/or pneumococcus.

### PM_2.__5_ Alters Microbiota Composition

**FIGURE 5:**
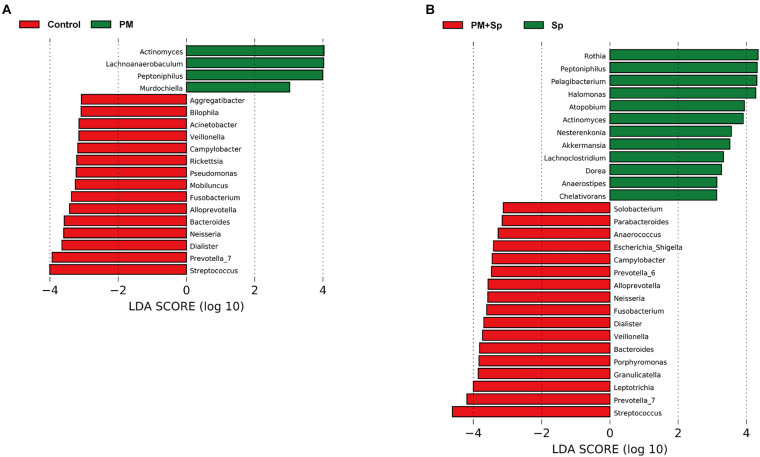
The differentially enriched microbiota genus in mice exposed to PM_2.__5_ and pneumococcus. LEfSe analysis showed abundance of bacterial genus (LDA > 3) was altered as compared between **(A)** control and PM_2.__5_ (PM); **(B)** pneumococcus (Sp) and PM_2.__5_ + pneumococcus (PM + Sp), respectively.

**FIGURE 6:**
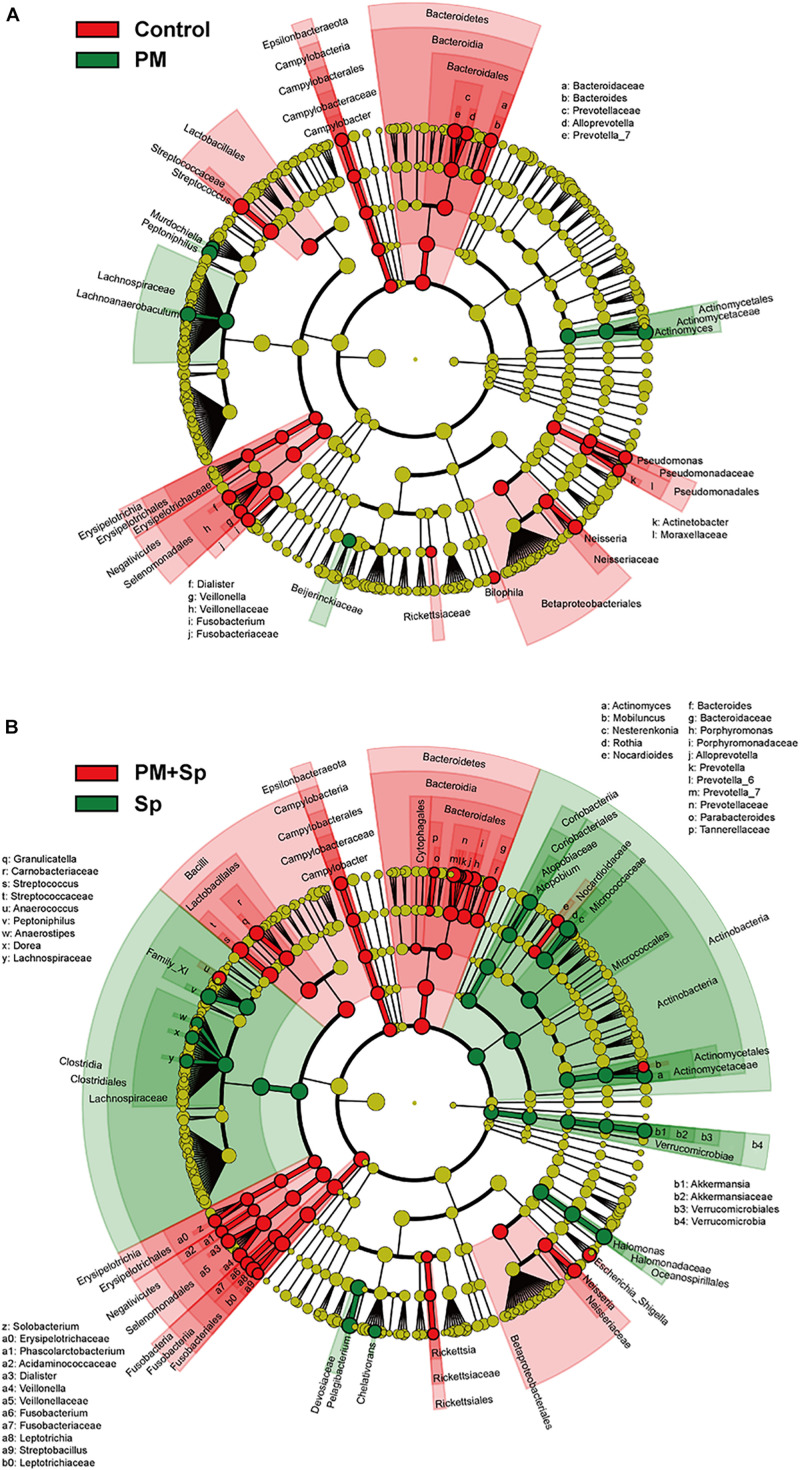
Circular taxonomic and phylogenetic trees of microbiota diversity. Comparison of the taxonomy for the PM_2.__5_-exposed in uninfected and pneumococcus-infected mice. The taxonomy was analyzed and performed as cladogram. Compared the effect of PM_2.__5_ altered microbiota composition in panels **(A)** control and **(B)** pneumococcus-infected (Sp) mice. The relative color represented the more abundance bacterial taxonomy in each group.

LEfSe analysis was performed to analyze the relatively enriched bacteria at genus and species levels among different groups. Many bacteria at the genus level were enriched in different groups; therefore, we selected a linear discriminant analysis (LDA) score higher than 3 or lower than -3 to represent the most significantly enriched genus in respective groups. As shown in Figure 5A, the relative abundances of *Actinomyces*, *Lachnoanaerobaculum*, *Peptoniphilus*, and *Murdochiella* were significantly increased in PM_2.__5_-exposed mice than in the control group. Notably, the relative abundance of *Streptococcus*, *Prevotella*, *Leptotrichia*, *Granulicatella*, *Porphyromonas*, and *Bacteroides* were mostly increased in mice co-treated with PM_2.__5_ and pneumococcus group than in the pneumococcus-infection alone group (Figure 5B). The specific enriched bacteria at the species level were then compared in PM_2.__5_-exposed to pneumococcus-uninfected or infected mice (Supplementary Figure 3). The significantly increased bacteria in the PM_2.__5_ + pneumococcus group were *Prevotella melaninogenica*, *Prevotella histicola*, *Veillonella dispar*, *Fusobacterium periodonticum*, and *Bacteroides coprocola* than in the pneumococcus-infection alone group. Moreover, the taxonomy and phylogenetic analysis were performed as cladogram. With PM_2.__5_ exposure, the microbiota richness was remarkably decreased than in the control group (Figure 6A). Furthermore, the enriched bacteria had notably different clusters in the PM_2.__5_ + pneumococcus group as compared to the pneumococcus-infection-alone group (Figure 6B). These results indicate that PM_2.__5_ exposure alters the microbiota composition and may increase the susceptibility of *Streptococcus* infection in mice.

### Functional Prediction of BALF Microbiota

**FIGURE 7:**
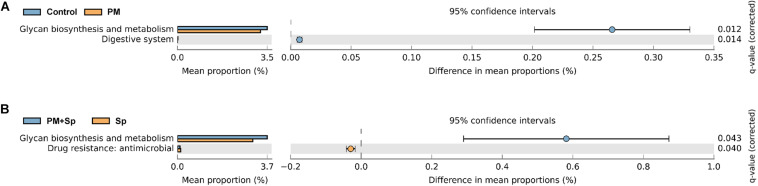
Putative ecological functions of bacterial communities altered by PM_2.__5_ and pneumococcus. The functions of bacterial communities were analyzed based on PICRUSt2 by KEGG annotation at level 2. Comparison of the functional profiles between **(A)** PM_2.__5_-exposed (PM) and unexposed (control) mice; **(B)** pneumococcal infection (Sp) and PM_2.__5_ plus pneumococcus (PM + Sp) infection.

To understand the influence of the microbiota community on disease progression, the functions of bacterial communities were analyzed using Phylogenetic Investigation of Communities by Reconstruction of Unobserved States 2 (PICRUSt2). Glycan biosynthesis, glycan degradation, lipopolysaccharide biosynthesis, biotin, vitamin B6, linoleic acid, and nitrogen metabolisms were associated with PM_2.__5_ exposure (Figure 7 and Supplementary Figure 4). Furthermore, the predominate pathways related to metabolisms such as glycan biosynthesis, glycan degradation, fatty acid biosynthesis, and several metabolisms were associated with PM_2.__5_ exposure followed by pneumococcal infection. These results indicate that differentially abundant features in mice treated with PM_2.__5_ and pneumococcus were correlated with host metabolisms, which may lead to disease development.

### PM_2.__5_ Exposure Combined With Pneumococcal Infection Exacerbated Pulmonary Pathogenesis

**FIGURE 8:**
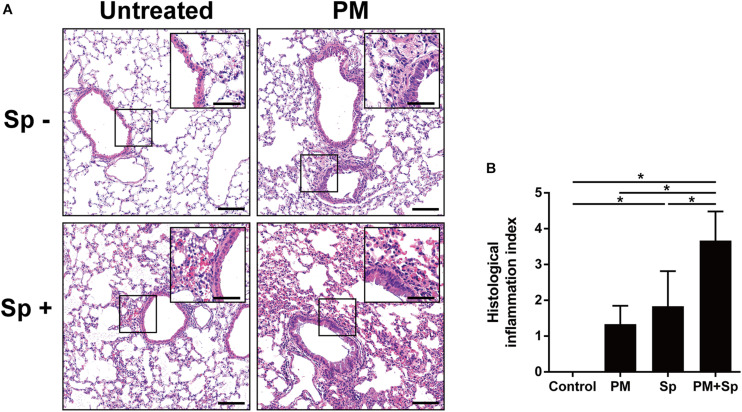
PM_2.__5_ exposure aggravated pneumococcus-induced lung inflammation. **(A)** Lung tissues were subjected to H&E staining. The magnified images were shown in the inset from each cropped area. **(B)** The stained tissues were examined and scored. PM, PM_2.__5_ treatment; Sp, pneumococcal infection. Scale bar in each panel, 100 μm, and in each inset, 50 μm.

To further analyze whether PM_2.__5_ enhanced pneumococcus-induced pathogenesis in the lungs, mouse pulmonary tissues were prepared for histopathological analysis. As shown in Figure 8A, the lung tissue sections from the control group showed a clearly defined epithelium with healthy alveoli. PM_2.__5_ exposure or pneumococcal infection caused pronounced inflammatory cell infiltration around the bronchi. Noticeably, by treating with PM_2.__5_ plus pneumococcus, enhanced inflammation and exuded erythrocytes in the pulmonary parenchyma were observed. Furthermore, significant bronchial wall thickening was observed in mice exposed to PM_2.__5_ combined with pneumococcal infection than that in either PM_2.__5_ or pneumococcal infection alone (Figure 8B). In addition, IHC analysis showed that the expression levels of iNOS and COX-2 were increased in PM_2.__5_ + pneumococcus mice compared with the control group (Supplementary Figure 5). The total bacterial survivals were increased in cells pretreated with PM_2.__5_ when compared to the untreated cells (Supplementary Figure 6). Collectively, our results demonstrate that PM_2.__5_ exposure alters the airway microbiota community and aggravates pneumococcus-induced lung pathogenesis.

## Discussion

PM_2.__5_ exposure is reported to change microbiota composition in respiratory and intestinal tracts (Mariani et al., 2018; Mutlu et al., 2018; Wang et al., 2018, 2019; Qin et al., 2019). However, the association between PM_2.__5_ and airway microbiota and its influence on pathogenic infection has not been investigated. In this study, we hypothesized that PM_2.__5_ interferes with the airway ecosystem to influence the bacterial community and disrupts pulmonary function, leading to increased susceptibility to pneumococcal infection. By using 16S rRNA sequencing analysis with Shannon-diversity index, observed ASVs, and Fisher’s diversity profiles, we observed that the abundance of BALF microbiota communities was decreased in both PM_2.__5_-exposure and PM_2.__5_ + pneumococcal infection groups than in control mice. It is known that resident microbiota contributes to host defense against pathogen infection (Liu et al., 2015). Once respiratory dysbiosis occurs, the fundamental roles performed by indigenous microbiota end up being modified, thereby leading to disease development (Marsland et al., 2013; Pascal et al., 2018). Intriguingly, the beta-diversity and cladogram indicated that the dominant community was similar in the control and PM + Sp groups. However, this restoration cannot be considered to suggest that the functions of microbiota were normal. Indeed, the histological analysis indicated severe pulmonary inflammation in mice with the combined treatment (PM + Sp). In addition, our recent study reported that macrophage functions were impaired by PM_2.__5_ exposure, which enhanced pneumococcal infectivity (Chen et al., 2020). These lines of evidence indicate that PM_2.__5_ alters the microbial ecosystem to benefit pneumococcus infection.

The predominant genera enriched in the PM_2.__5_ + pneumococcus group were *Streptococcus*, *Prevotella*, *Leptotrichia*, *Granulicatella*, *Porphyromonas*, and *Bacteroides*, which are involved in glycan biosynthesis and metabolism in compliance with the results from PICRUSt2 analysis (Thompson and Pikis, 2012; Zhou et al., 2016; Emerson and Weimer, 2017; Robinson et al., 2017). The bacterial cell wall is coated with diverse glycoproteins that help respond to the environment. They participate in biological processes like virulence and are linked to pathogenesis in the progression of many diseases (Tra and Dube, 2014; Williams et al., 2020). Intriguingly, the glycosylation process in *Streptococcus* is associated with the ability of adherence to the host and taking part in immune evasion (Schmidt et al., 2003). These findings indicated that glycan biosynthesis and metabolism are closely related to bacterial infectivity and pathogenesis. However, the mechanism underlying how the glycosylation process senses environmental factors requires further exploration.

The genera observed in our study were similar to those observed in humans who smoked and were exposed to air pollution (Hosgood et al., 2014; Shen et al., 2016; Li et al., 2019; Qin et al., 2019). Chronic PM_2.__5_ exposure altered microbiota richness and induced abnormalities in glucose homeostasis (Li et al., 2020), which correlated with diabetes development (Liu et al., 2018, 2019). Although these studies revealed the link between PM_2.__5_ and glucose-metabolic effects, the underlying mechanism of microbiota in the regulation of host glucose homeostasis needs further investigations.

Short-chain fatty acids and lipid metabolites produced from commensal microbiota are shown to impair the inflammatory responses and promote disease development, including asthma, colitis, and intestine atherosclerosis (Maslowski et al., 2009). Moreover, the recognition of commensal bacteria-derived products by toll-like receptors is an important line in intestine homeostasis (Rakoff-Nahoum et al., 2004). These findings indicate the importance of the metabolites in influencing the commensal microbiota to cause diseases. In addition to regulating metabolism, our study also calls attention to the role of the modulation in resident bacterial genera in infectious disease and pathogenesis. For instance, *Streptococcus*, a Gram-positive coccus, is the most common cause of human respiratory diseases and several diseases are associated with *S. pneumoniae* infection, including bacteremia, meningitis, and pneumonia, a lower respiratory tract disease (Wardlaw et al., 2006). Notably, *Streptococcus* was abundant in patients infected with *Mycobacterium tuberculosis* and influenza virus (De Lastours et al., 2016; Ding et al., 2019; Eshetie and van Soolingen, 2019). For genera *Bacteroides* and *Prevotella*, which are commonly observed in Gram-negative lung microbiota, a recent study demonstrated their role in promoting fibrotic pathogenesis through their outer membrane vesicle-mediated IL-17R signaling (Yang et al., 2019). In addition, *Leptotrichia* has been found in BALF as a potentially causative bacterium for severe pneumonia (Kawanami et al., 2009). These lines of evidence indicate that PM_2.__5_ alters the microbiota community and particularly enhances the clinically important bacteria, which may collectively participate in exacerbating lung pathogenesis.

Macrophages play a crucial role in the clearance of pathogens. We recently demonstrated that PM_2.__5_ adversely influences macrophage activity to enhance pneumococcal infectivity and exacerbate pulmonary pathogenesis (Chen et al., 2020). Our results showed that PM_2.__5_ impairs macrophage functions, such as phagocytosis and cytokine production, resulting in reduction in its bacterial clearance activity. In addition, PM_2.__5_ manipulates macrophage polarization by suppressing M1 macrophage markers and then subverts macrophage defense against bacterial infection, thereby aggravating inflammation. It is worth noting that PM_2.__5_ exposure altered the microbiota community and led to disease progression (Mariani et al., 2018; Qin et al., 2019). Although the commensal microbiota can protect from asthma and allergic responses (Kirjavainen et al., 2019), an aberrant microbiota community is correlated with airway diseases (Hoggard et al., 2017, 2018). Along with the results of previous reports, the outcomes of our present study reveal that PM_2.__5_ affects both immune defense and microbiota dysbiosis, thereby exacerbating pathogen infection in the respiratory tract.

Although this study provides evidence that PM_2.__5_ exposure-altered airway microbiota is associated with an increased susceptibility of pneumococcal infection, some limitations exist. First, the sample size of each treatment group is small in the LEfSe analysis for characterizing the changed microbiota composition among different experimental groups. The features of LEfSe analysis included the tests of biological consistency and effect-size estimation, particularly given the small number of test samples (Segata et al., 2011; Goodrich et al., 2014). The inclusion of more mice in the future studies was required to reduce any bias in the sequencing analysis. Second, the detailed mechanism underlying the PM_2.__5_-altered microbiota community to induce pathogenesis was not explored in the current study. Third, the pathogenic patterns and PM_2.__5_ natural exposure ways are different between mice and human, and hence, a direct clinical study merits further investigation. Therefore, further studies of the samples isolated from patients with pneumococcal infection living in PM_2.__5_-exposed areas are required to support the clinical manifestations. Since the composition of indigenous microbiota is quite complicated and plays crucial roles in the regulation of human immunity, physiology, and metabolism, systemic human studies with extensive investigation of airway microbiota and the ecosystems in long-term exposure to PM_2.__5_ are warranted.

## Conclusion

In summary, our results showed that PM_2.__5_ exposure is associated with a decrease of lung microbiota diversity. Further, the microbiota community is dynamically altered in response to PM_2.__5_ exposure followed by pneumococcal infection. The results from this study provide a better understanding of the adverse effects of PM_2.__5_ on the pulmonary microbiota community, which may enhance pathogen infection and aggravate lung pathogenesis.

## Data Availability Statement

The datasets presented in this study can be found in online repositories. The names of the repository/repositories and accession number(s) can be found in the article/Supplementary Material.

## Ethics Statement

The animal study was reviewed and approved by Institutional Animal Care Use Committee, Chang Gung University (IACUC Approval No.: CGU16-019).

## Author Contributions

C-DL, C-HC, C-YY, and C-HL: conception or design of this work. Y-WC, S-WL, M-ZH, H-JL, and C-YC: experimental study. Y-WC, S-WL, and Y-RL: data analysis and interpretation. Y-WC, S-WL, C-DL, C-HC, C-YY, and C-HL: writing the manuscript. All authors contributed to the article and approved the submitted version.

## Conflict of Interest

The authors declare that the research was conducted in the absence of any commercial or financial relationships that could be construed as a potential conflict of interest.

## Footnotes

1 http://www.ncbi.nlm.nih.gov/bioproject/661979

## Supplementary Material

Supplementary Figure 1 Comparison of BALF microbiota diversity. PCoA was conducted to analyze the BALF microbiota compositions in mice treated with control (red), PM_2__.5_ (PM, green), pneumococcus (Sp, purple), PM_2__.5_ + pneumococcus (PM + Sp, blue).

Supplementary Figure 2 Relative abundance of bacterial genera in mice exposed to PM_2__.5_ and pneumococcus. The bacteria taxonomic profiles at genus level in BALF microbiota from PM_2__.5_-treated and/or pneumococcus-infected mice. Relative abundance of **(A)** *Halomonas*, **(B)** *Ezakiella*, **(C)** *Granulicatella*, **(D)** *Staphylococcus*, **(E)** *Atopobium*, **(F)** *Haemophilus*, **(G)** *Prevotella*, and **(H)** *Megamonas* in BALF microbiota were analyzed. Statistical significance was analyzed using the Student’s *t*-test. ^∗^*P* < 0.05; ^∗∗^*P* < 0.01; ^∗∗∗^*P* < 0.001; ^∗∗∗^*P* < 0.0001. PM, PM_2__.5_ exposure; Sp, pneumococcal infection.

Supplementary Figure 3 The differentially enriched bacterial species compared with PM_2__.5_ exposure and pneumococcus-infected mice. LEfSe analysis indicated the abundance bacterial species compared with **(A)** control and PM_2__.5_ exposure (PM); **(B)** pneumococcus (Sp) and PM_2__.5_ + pneumococcus (PM + Sp).

Supplementary Figure 4 Putative functions of microbiota communities altered by PM_2__.5_ and pneumococcus. The functions of microbiota were predicted based PICRUSt2 by KEGG annotation at level 3. **(A)** Comparison of the functional profiles between **(A)** control and PM_2__.5_ exposure (PM); **(B)** pneumococcus (Sp) and PM_2__.5_ + pneumococcus (PM + Sp).

Supplementary Figure 5 PM_2__.5_ exposure aggravated pneumococcus-induced pulmonary inflammation. Mice were euthanized and lung tissues were subjected to immunohistochemical (IHC) staining with specific antibody against iNOS and COX-2 (original magnification: 200×). The magnified images are shown in the panel of each cropped area. Scale bar in each panel, 100 μm, and in each inset, 50 μm.

Supplementary Figure 6 PM_2__.5_ suppresses macrophage clearance to increase bacterial survival. RAW264.7 cells were exposed to low (5 μg/ml) or high (20 μg/ml) doses of PM_2__.5_ for 2 h, and then incubated with and pneumococcus (MOI = 100) for the indication times. Total pneumococcal survival was determined and expressed as viable CFU. Results are represented mean ± standard deviation from triplicate independent experiments. Statistical significance was analyzed using the Student’s *t*-test (^∗^*P* < 0.05 compared to PM_2__.5_-untreated group).
