# Supplementary figures and images for: Fine Particulate Matter Exposure Alters Pulmonary Microbiota Composition and Aggravates Pneumococcus-Induced Lung Pathogenesis
Source: Front Cell Dev Biol. 2020 Oct 26;8:570484. doi: 10.3389/fcell.2020.570484 (PMC7649221; doi:10.3389/fcell.2020.570484)

Fig. S1

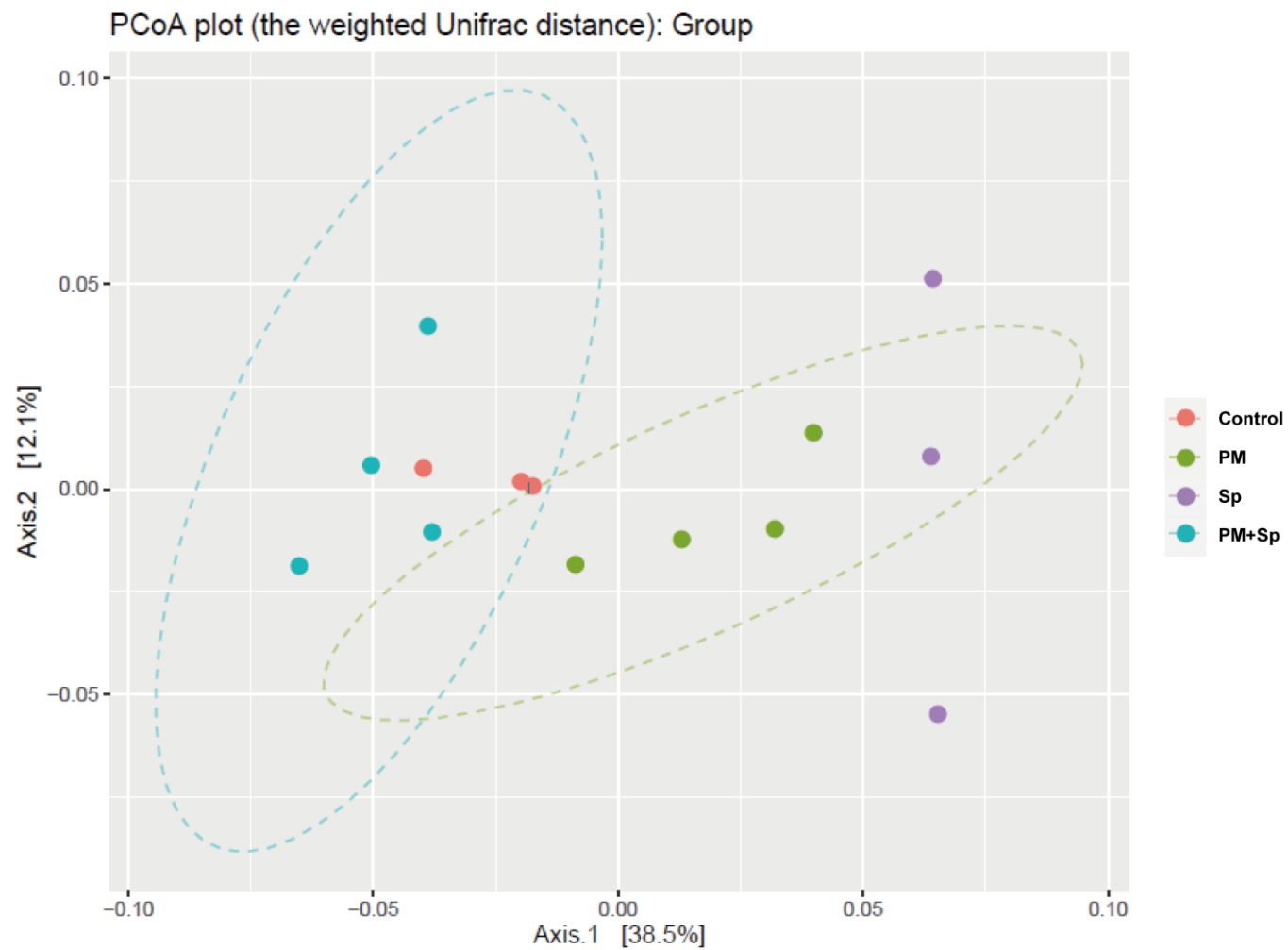

Fig. S2

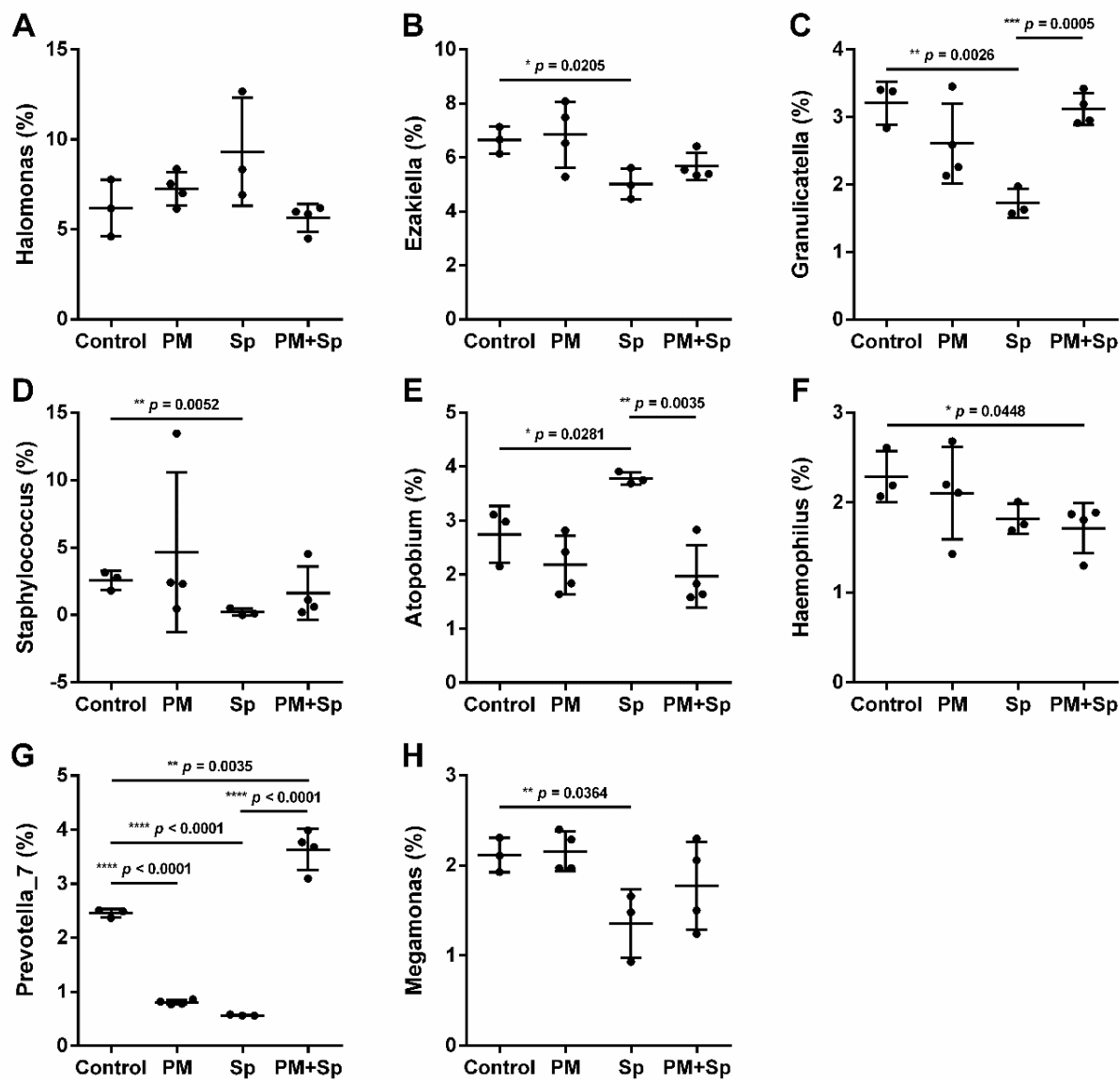

Fig. S3

**A**

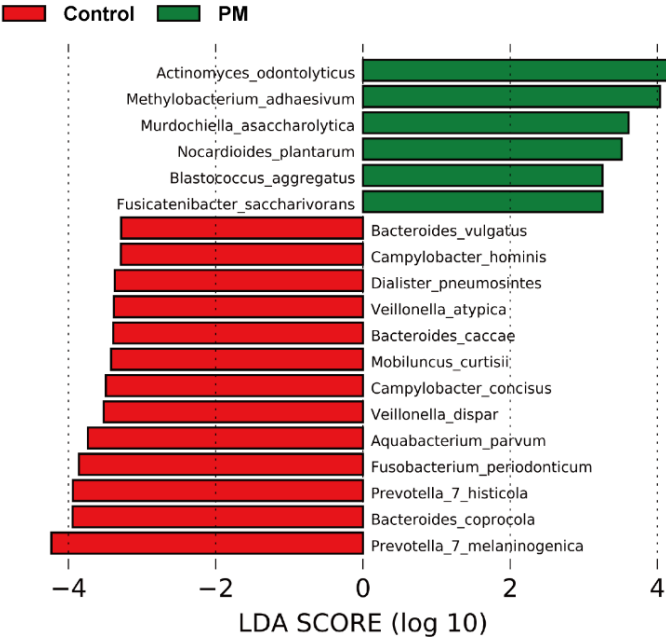

**B**

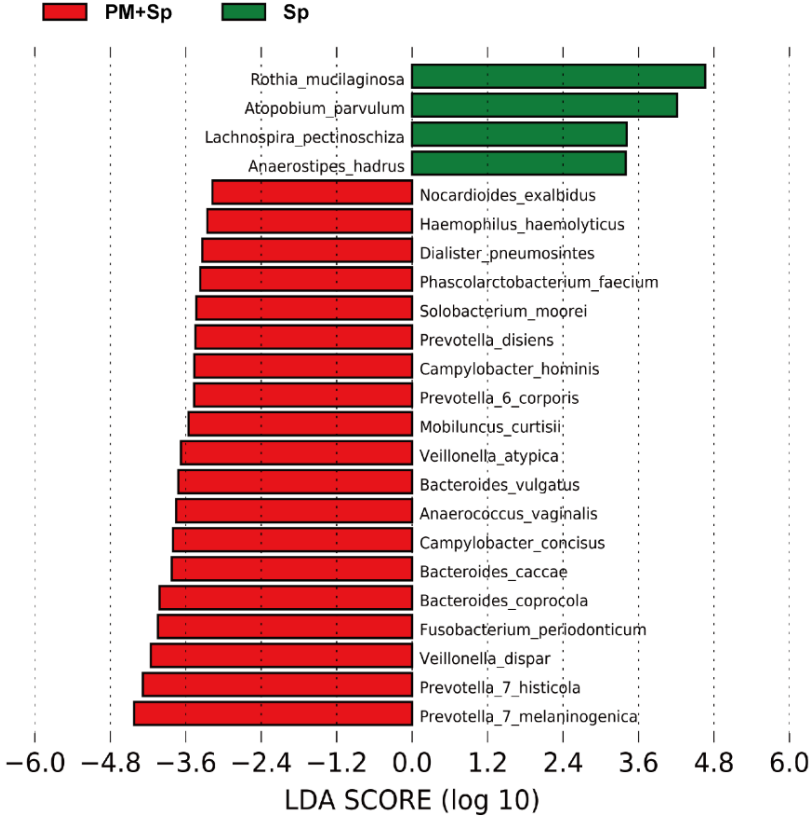

Fig. S4

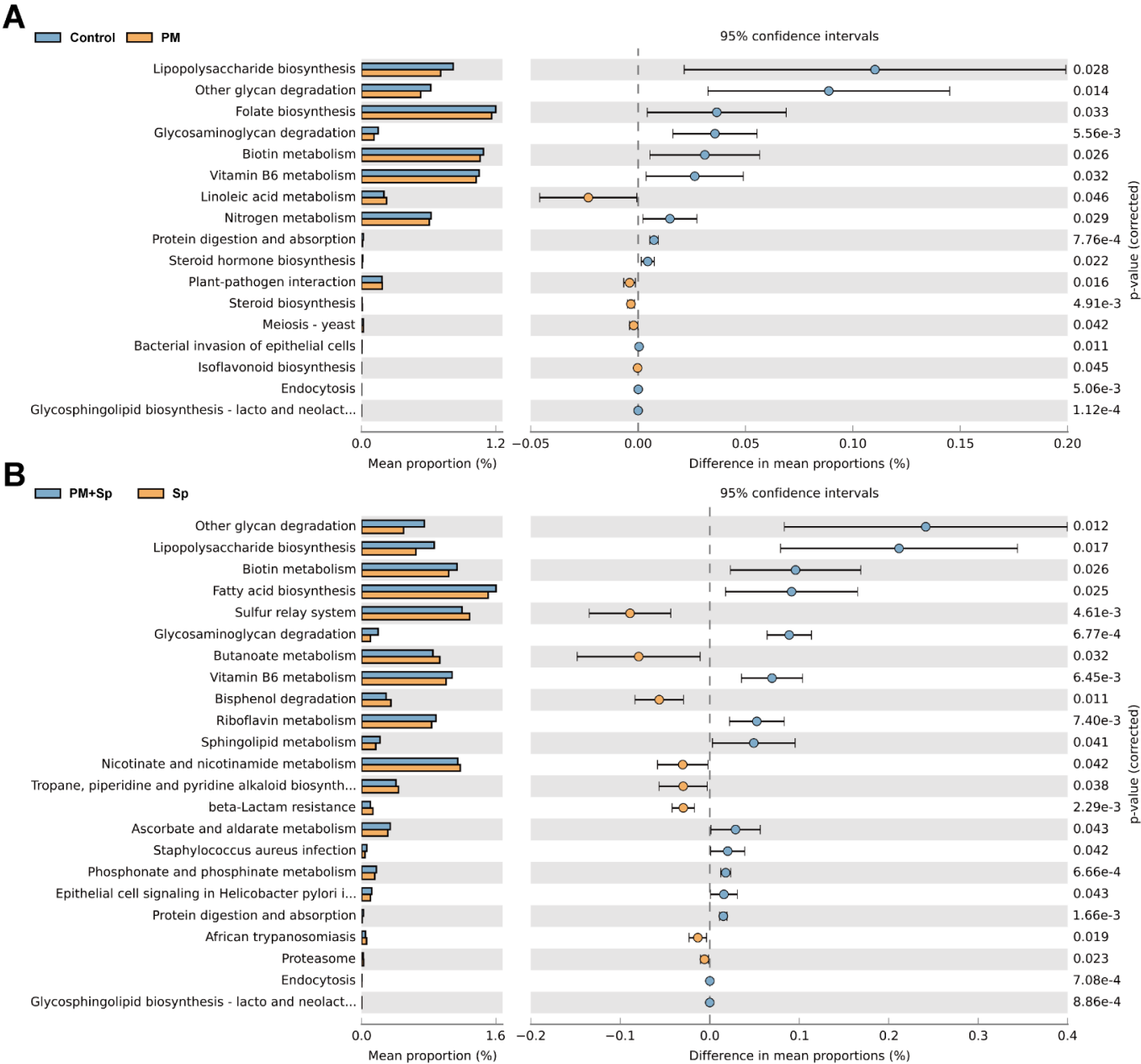

Fig. S5

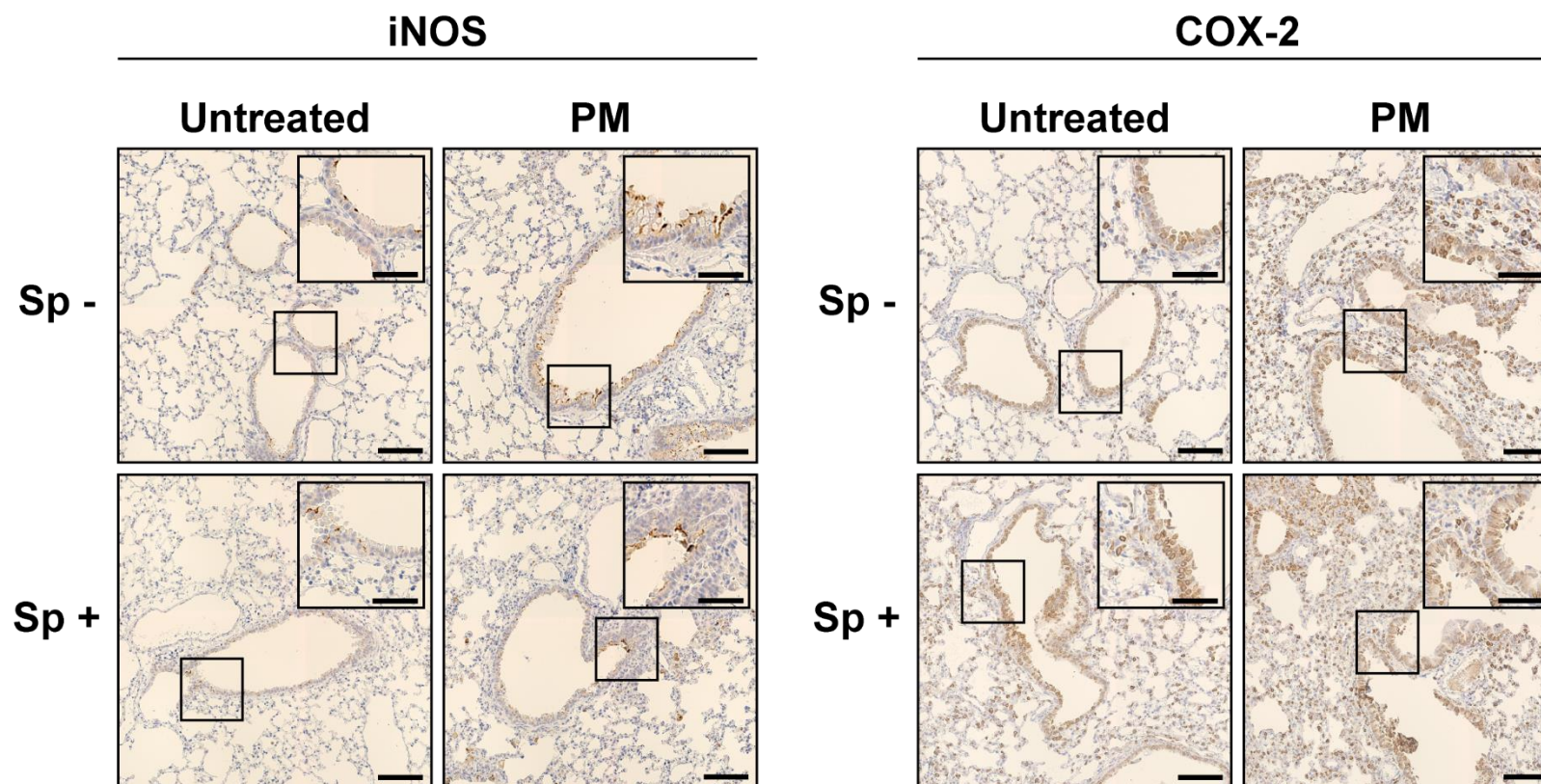

Fig. S6

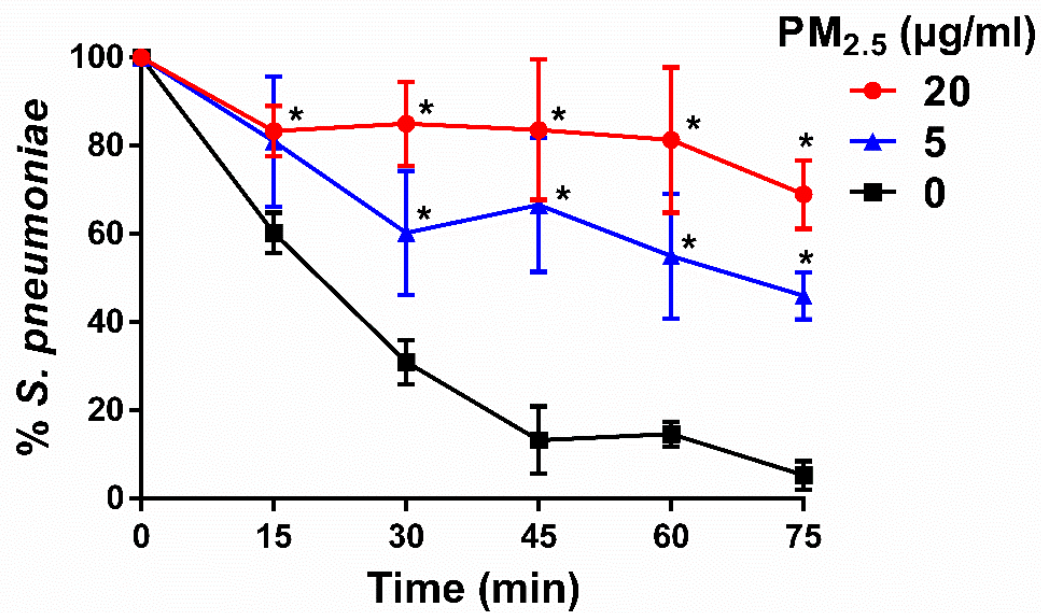

Supplement: Supplementary Figure 1 — Comparison of BALF microbiota diversity. PCoA was conducted to analyze the BALF microbiota compositions in mice treated with control (red), PM2.5 (PM, green), pneumococcus (Sp, purple), PM2.5 + pneumococcus (PM + Sp, blue). [file Presentation_1.pdf]
